# Nepal: self-reliant in ophthalmic human resources

**Published:** 2018-07-31

**Authors:** Sanjay Kumar Singh, Sudhir Thakur, Afaque Anwar

**Affiliations:** 1 Programme Director: Eastern Regional Eye Care Programme, Biratnagar, Nepal.; 2 Programme Coordinator: Eastern Regional Eye Care Programme, Biratnagar, Nepal.; 3 Health Educator: Biratnagar Eye Hospital, Biratnagar, Nepal.

**In the early 1980s, Nepal barely had any eye care professionals – neither ophthalmologists nor ophthalmic assistants. In three decades this was addressed systematically and Nepal now has a significant workforce, adequate in-country training capacity and training ophthalmologists for other developing countries in Asia. Nepal's incredible journey is an inspiration for other developing countries.**

**Figure:**
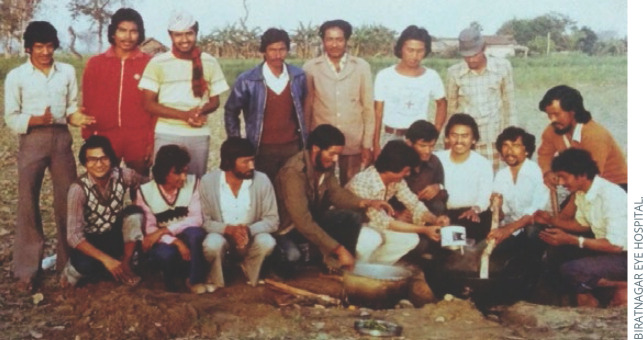
First batch of ophthalmic assistants in Nepal in 1981. NEPAL

The VISION 2020: The Right to Sight global initiative emphasised the key role that eye care human resources play in reducing avoidable blindness, both at a national and global level.^1^ In a short period, Nepal has achieved remarkable progress in reducing avoidable blindness and developing a formidable eye care work force. This article takes you through Nepal's journey in becoming self-reliant in ophthalmic human resources.

In the 1980s, Nepal had only one eye hospital in the capital city, Kathmandu, and seven ophthalmologists in the urban centers of Central and Eastern regions and no other trained eye care workforce. About 1000 cataract surgeries were performed every year as people would often travel to India for cataract surgeries. There were no training programmes, either for ophthalmologists or paramedical staff.^2^

In 1978, nine enterprising and passionate individuals, comprising of social workers, ophthalmologists, industrialists and traders started the Nepal Netra Jyoti Sangh (NNJS). It was started as a national society to develop and provide high quality, sustainable, comprehensive and affordable eye care services to the people of Nepal.

A national blindness survey in 1980-1981 showed the prevalence, distribution and causes of blindness in Nepal. The survey helped in formulating a national plan for the development of eye care services and reduction of avoidable blindness. One of the key components of the plan was to be self-reliant in the ophthalmic human resources within the next 20 years. To achieve that target NNJS reached out to NGOs, hospitals and different institutes in India and other countries to provide post-graduate training in ophthalmology to students from Nepal.

- 10 medical doctors in different batches received training from the All India Institute of Medical Sciences (AIIMS) in New Delhi.
- Three doctors received training from Post Graduate Institute of Medical Research (PGIMR), Chandigarh and five doctors received training from Kasturba Medical College (KMC), Manipal. Different organisations like the World Health Organization (WHO) and the Swiss Red Cross financially supported the training of these ophthalmologists.
- Similarly, six doctors received training from the United Kingdom through the British Council.

**Figure 1:**
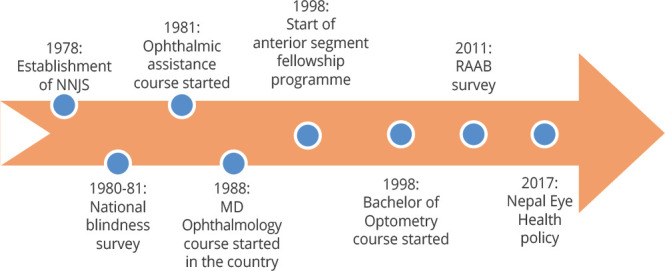
Timeline of key events in Nepal's journey

## 1988: post graduate MD ophthalmology course in Nepal

In Nepal, a post-graduate course in ophthalmology was started in 1988 at the Tribhuvan University Teaching Hospital. Initially, only two candidates were enrolled per year and this was later increased to ten. After the establishment of the National Academy of Medical Sciences (NAMS), existing infrastructure and human resources of different eye hospitals began to be utilised through NAMS for post-graduate training. Presently through different institutes, 45 ophthalmologists are trained each year in Nepal.

Fellowship in anterior segment surgeries was started at Sagarmatha Choudhary Eye Hospital, Lahan in 1998 to attract fresh and young ophthalmologists to come and support the existing workload. Different subspecialty fellowships were also started in Tilganga Institute of Ophthalmology and other eye hospitals. At present subspecialty fellowship training is available in almost all ophthalmic institutes.

## 1981: ophthalmic assistant course

To deal with the acute shortage of paramedical staff, a three-year ophthalmic assistant training course was started in 1981 and 50 ophthalmic assistants were enrolled in first batch. The main objective of this course was to develop multitasking paramedical staff to assist ophthalmologists in managing patients at out-patient departments, operation theatres and camp settings. Ophthalmic assistants received training in diagnosis, management and treatment of common ophthalmic disorders. They were also trained to assist in operations, in giving retrobulbar anaesthesia and perform minor eye operations. Currently, eight eye hospitals in affiliation with the Council for Technical Education and Vocational Training (CTEVT) train about 320 ophthalmic assistants each year. As of today, there are 920 ophthalmic assistants and about 600 more are required by the year 2020.

## 1998: bachelor in optometry

A bachelors in optometry course was started for the first time in 1998 at the B P Koirala Lions Centre for Ophthalmic Studies. Recently, bachelors' courses in optometry and vision science was started at different eye hospitals in affiliation with NAMS. At present, 50 optometrists receive training every year from two institutes and their affiliated hospitals. Another 50 optometrists trained in India and other countries also join the eye health workforce every year. There are 350 optometrists in Nepal and nearly 250 more are needed by the year 2020.

**Figure 2:**
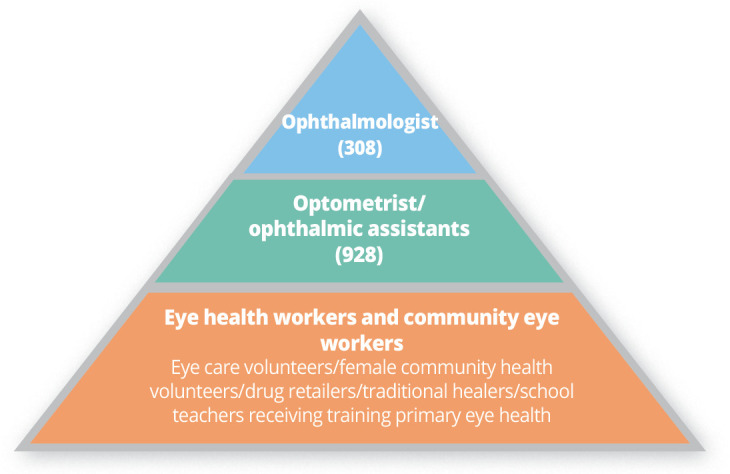
Three tier pyramid of ophthalmic human resource in Nepal

## Eye health worker and community eye workers

Community eye workers form a strong referral network for patients seeking eye care within their communities to the eye care centres and eye hospitals. Eye health workers carry out numerous tasks while aiding ophthalmologists, ophthalmic assistants and optometrists in operation theatres, out-patient departments, for optical dispensing, and screening and surgical eye camps. Various eye hospitals in affiliation with CTEVT are running this training programme for community eye workers as per the need.

Most of the eye hospitals in Nepal provide a one day to one week training on primary eye care to eye care volunteers, female community health volunteers, drug retailers, school teachers and traditional healers. According to their background, different training modules of varying duration are designed for these volunteers. Although the available ophthalmic human resources do not meet the required number according to WHO standards, the capacity is increasing gradually to cater to the needs of the Nepalese population. Despite having insufficient number, more than 3 million patients were examined and more than 300,000 operations were performed in the year 2017. This is only possible due to adequate and effective utilisation of existing human resources.

## Challenges

**Brain Drain:** A mid-term VISION 2020 review in 2010 showed that brain drain was a major challenge in terms of human resources. Nearly 36% of optometrists, 25% of ophthalmic assistants and 11.2% of ophthalmologists moved out of Nepal for better opportunities.

**Distribution:** There is an inequality in the distribution of existing human resources in Nepal. Geographically all ophthalmologists are positioned in hilly and flat areas of Nepal whereas not a single ophthalmologist practices in the mountainous region. Nearly 37% of population in Provinces 1 and 3 have access to 60% of eye care human resources while the remaining 63% are served by 40% human resources.

**Figure 3:**
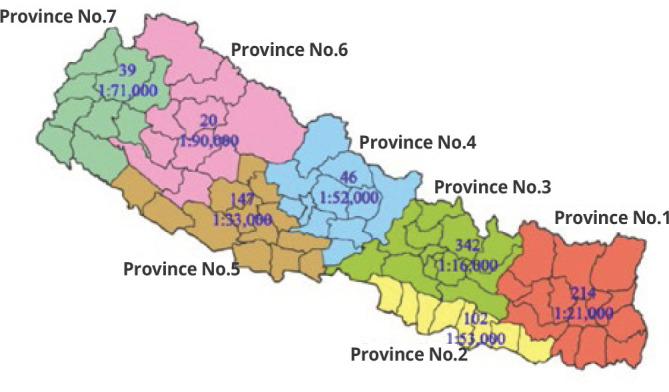
Distribution of eye care human resources in Nepal

**Lack of job opportunities in government health care system:** In Nepal, non-governmental organisations (NGO) and privately-run eye hospitals provide basic eye care to tertiary level services throughout the country. This has led to under-utilisation of existing government infrastructure in rural and urban areas.

**Insufficient number of trained human resources with different subspecialties:** The rapid assessment of avoidable blindness (RAAB) survey done in 2010 showed that cataract is the major cause of blindness followed by retinal disease, glaucoma and corneal disease. Nepal has insufficient number of specialists to deal with new emerging causes of blindness.

## Conclusion and recommendations

Although there has been tremendous progress in availability of trained ophthalmic human resources, there is a need for more, to meet future challenges. Nepal needs to address a gap in specialists and other eye health professionals. Inequality in the distribution of human resources in different states and across different geographical regions can be tackled by providing extra incentives and opportunities for continuous medical education. We need to provide opportunities for ophthalmic human resources to work within government systems, so that existing HR can be distributed at community and district levels in different geographical regions of Nepal. Furthermore, qualitative and quantitative research is needed to test innovative ways to recruit and retain the work force.
